# Long-Term Outcomes in First-Episode Psychosis: Insights from a 20-Year Cohort Study to Inform Recovery-Oriented Care Models

**DOI:** 10.1192/j.eurpsy.2025.130

**Published:** 2025-08-26

**Authors:** M. N. Clarke

**Affiliations:** Psychiatry, St John of God Community Mental Health Services, Dublin, Ireland

## Abstract

**Background:**

Cohort studies in first-episode psychosis (FEP) provide crucial insights into the diverse trajectories of clinical and functional recovery. These studies are invaluable for evaluating the effectiveness of new intervention models and informing resource allocation and policy development.

**Objectives:**

To synthesize quantitative and qualitative findings on mortality and clinical outcomes and to explore their interrelations in a multi-modal investigation of long-term outcomes in FEP. Specific objectives include appraising how this study’s findings have influenced the development of new models of care.

**Methods:**

Data were drawn from the iHOPE-20 study, comprising cohort analyses and qualitative interviews with 171 FEP participants diagnosed between 1995–1999 in Dublin, Ireland. Participants with lived experience contributed to the design of the 20-year follow up assessments. Mortality rates were calculated; symptoms, functionality and quality of life trajectories were analyzed using mixed models; and personal recovery themes were derived through thematic analysis. Ongoing analyses are addressing predictors of the number, timing and sequencing of relapses/readmissions among baseline variables as foci for service development.

**Results:**

The study revealed substantial variation in long-term outcomes among individuals with FEP. Shorter DUP was consistently associated with better outcomes across all of the follow-up points. Mortality rates highlighted the importance of interventions to address physical health morbidity. Diagnostic instability over time underscored the dynamic nature of psychosis management. Employment status at follow-up highlighted the importance of strategies to support a return to education or employment after a first presentation.

**Conclusions:**

Findings emphasize the enduring impact of DUP and the need for tailored interventions. Data from this cohort highlight the value of longitudinal insights as a benchmark for comparing the effectiveness of new intervention models. In such studies, addressing ethical and data protection challenges, incorporating the expertise of those with lived experience and harmonising outcome measures remain vital to advance recovery-oriented care models.

**Disclosure of Interest:**

None Declared

